# Supplementary material for: RNA Interference-Mediated Suppression of Ecdysone Signaling Inhibits Choriogenesis in Two Coleoptera Species
Source: Int J Mol Sci. 2024 Apr 22;25(8):4555. doi: 10.3390/ijms25084555 (PMC11050585; doi:10.3390/ijms25084555)
Supplement: Supplementary file 1 [file ijms-25-04555-s001.zip › ijms-2938131-supplementary.pdf]

Supplementary data

RNA Interference-Mediated Suppression of Ecdysone Signaling Inhibits Choriogenesis in two Coleoptera species

Xiao-Qing Zhang [1], Lin Jin [1], Kai-Yun Fu [2, 3], Wen-Chao Guo [2, 3], Guo-Qing Li [1] *

1 Education Ministry Key Laboratory of Integrated Management of Crop Diseases and Pests/ State & Local Joint Engineering Research Center of Green Pesticide Invention and Application, Department of Entomology, College of Plant Protection, Nanjing Agricultural University, China

2 Key Laboratory of Intergraded Pest Management on Crops in Northwestern Oasis, Ministry of Agriculture, China

3 Xinjiang Key Laboratory of Agricultural Biosafety, Institute of Plant Protection, Xinjiang Academy of Agricultural Sciences, Urumqi, China

Table S1. Primers used in RT-PCR, dsRNA synthesis and qPCR

Fragment name	Forward primer	Reverse primer
**dsRNA synthesis**		
ds*HvEcR*	ACAAAGTGATGTCAGATTTAG	GCATTATCTACTTTCATGTAA
ds*Hvusp*	CTTATCCACCCAACCATC	CTTCTCTTTTCATACCCATC
ds*LdEcR*	GCAGCATGGACATCAAACAC	GCACTTACACTAATGGCTCCC
ds*Ldusp*	AATCAATAAGTCCACCGC	CCAGACACTTCAAACCGA
ds*egfp*	AAGTTCAGCGTGTCCG	CACCTTGATGCCGTTC
**qPCR**		
q*LdEcR*	GAATGAGGGCAGAGTGTGTG	TCGTAGTGCTATTGGGCTTG
q*LdEcRA*	CGGGTTCAATAACAGTGTCG	ATAAGGTTGCGAAGGTGGTC
q*LdEcRB1*	GGGAGTGCTAGTGTTGTGGA	GGATTAGACGCTCCCTACGA
q*Ldusp*	GTACTGGCATCTGTTTCTTTGTCG	CAAGAAGAACGGCAAAGGACGA
q*LdUAP1*	AGAGCTGGAGGCGTAGTTGT	TTTGATTGTTCGGATTTGGA
q*LdChS1*	TTGGAACCATAGCTCACATCTT	AATCTCCACTGCCTGCTTATC
q*LdChS2*	AGACTTCTGGTGTTGCTCTTC	GTAGGCGCATTCGTCCTTAT
qLdRP18	TAGAATCCTCAAAGCAGGTGGCGA	AGCTGGACCAAAGTGTTTCACTGC
qLdRP4	AAAGAAACGAGCATTGCCCTTCCG	TTGTCGCTGACACTGTAGGGTTGA
qHvEcR	AGAAGAAAGCCTAGGTCCGG	GTCCCATATCTCGGCCAAGA
qHvEcRA	GCGTAGATGGACGGGTATGC	AACGTCGCAGACGAAGATGT
qHvEcRB1	GCAGCTCCAATCTCAATGGT	GAATTCGCGTTCTGGTTGTT
qHvusp	GGATGCATCAGAGGTGGAGT	AAACTTGGTCACACACTGGC
q*HvUAP*	CACAGCTAAGCGCGACATTC	CTCCAGTTCTTCGCCACTGT
q*HvChS1*	CTGTCGATCCAACCGGACAT	CTTCGGCTAGTGGTCCAAGG
q*HvChS2*	TTGGCTGCAAAAAGCGACTG	GATGTCTAGCTTCGGACGCA
qHvRPS18	CGCAATCAAAGGTGTTGGAAG	GCCTAGGGTTGGCCATAATAG
qHvRPL13	AGCATCCTTCGCTCGTTTAG	TTCGACAACCTGCCATTAGG
